# Electron donation of non-oxide supports boosts O_2_ activation on nano-platinum catalysts

**DOI:** 10.1038/s41467-021-22946-y

**Published:** 2021-05-12

**Authors:** Tao Gan, Jingxiu Yang, David Morris, Xuefeng Chu, Peng Zhang, Wenxiang Zhang, Yongcun Zou, Wenfu Yan, Su-Huai Wei, Gang Liu

**Affiliations:** 1 State Key Laboratory of Inorganic Synthesis and Preparative Chemistry, College of Chemistry, Electron Microscopy Center, Jilin University, Changchun, China; 2 Key Laboratory for Comprehensive Energy Saving of Cold Regions Architecture of Ministry of Education, School of Materials Science and Engineering, Jilin Jianzhu University, Changchun, China; 3 Department of Chemistry, Dalhousie University, Halifax, Canada; 4 Jilin Provincial Key Laboratory of Architectural Electricity & Comprehensive Energy Saving, School of Electrical and Electronic Information Engineering, Jilin Jianzhu University, Changchun, China; 5 Beijing Computational Science Research Center, Beijing, China

**Keywords:** Atmospheric chemistry, Heterogeneous catalysis, Atmospheric chemistry, Materials for energy and catalysis

## Abstract

Activation of O_2_ is a critical step in heterogeneous catalytic oxidation. Here, the concept of increased electron donors induced by nitrogen vacancy is adopted to propose an efficient strategy to develop highly active and stable catalysts for molecular O_2_ activation. Carbon nitride with nitrogen vacancies is prepared to serve as a support as well as electron sink to construct a synergistic catalyst with Pt nanoparticles. Extensive characterizations combined with the first-principles calculations reveal that nitrogen vacancies with excess electrons could effectively stabilize metallic Pt nanoparticles by strong p-d coupling. The Pt atoms and the dangling carbon atoms surround the vacancy can synergistically donate electrons to the antibonding orbital of the adsorbed O_2_. This synergistic catalyst shows great enhancement of catalytic performance and durability in toluene oxidation. The introduction of electron-rich non-oxide substrate is an innovative strategy to develop active Pt-based oxidation catalysts, which could be conceivably extended to a variety of metal-based catalysts for catalytic oxidation.

## Abstract (web summary)

Activation of O_2_ is a critical step in heterogeneous catalytic oxidation. Here, the authors adopt the concept of increased electron donors induced by nitrogen vacancy to develop an efficient strategy for preparing highly active and stable catalysts for molecular O_2_ activation.

## Introduction

Catalytic oxidation encompasses a great deal of important processes in the chemical industry and emissions control, such as ethylene epoxidation, CO, and hydrocarbon conversion^1–5^. These processes account for a considerable amount of global chemical production, especially the energy consumption reactions^2^. Platinum is a widely used metal reactive centers of catalysts for emissions control^4–6^. Considering the cost of the catalysts and energy consumption of the reaction, one desires to achieve complete conversion of pollutants at the lowest possible reaction temperature with lowest amount of Pt loading^7–9^.

Activation of molecular oxygen is a critical step for heterogeneous catalytic oxidation^2,10–12^. The reactivity of O_2_ adsorption and dissociation (two successive steps in O_2_ activation) on the catalyst surface relies on the charge transfer from the metal surface, and/or metal-support interface to the stable O_2_ molecules^2,10^. Sufficient charge transfer would weaken the O–O bond and render it more reactive toward reactant molecules^2^. Therefore, introducing excess electrons to the Pt centers and/or the Pt-support interface should be a practical strategy to obtain a highly active Pt-based catalyst.

Oxides materials are widely adopted as supports to stabilize the Pt nanoparticles^13–18^. Surface oxygen vacancies could serve as electron donation sites to enhance the performance of O_2_ activation^19^. However, the metallicity of Pt nanoparticles inevitably decrease during the loading process onto the oxides support^20^. The solid-state reaction between Pt nanoparticles and the oxide support cause the oxidation of the neutral Pt atoms to cationic atoms and this, in turn, can weaken the charge transfer for O_2_ activation^8^.

As a non-oxide and metal-free material, carbon nitride (especially g-C_3_N_4_) has become a star material in areas of photocatalysis and electrocatalysis in the past decade^21–26^. Basically, carbon nitride is a class of polymeric materials consisting mainly of carbon and nitrogen. The conjugated polymeric network endows carbon nitride with desirable physicochemical properties, such as insolubility in acidic, neutral, or basic solvents^26^. In comparison with conventional carbon materials, inertness against oxidation is an important feature of carbon nitride^22^. However, this attractive property negatively affects their performance in heterogeneous catalysis. The inert surface renders it less reactive toward reactants and few active heterogeneous oxidation catalysts have been fabricated based on carbon nitride materials.

In this work, the potential advantage of carbon nitride was fully amplified through manipulating the nitrogen-vacancy of carbon nitride. The increased electron donors induced by nitrogen vacancy was demonstrated as an efficient strategy to develop highly active and stable Pt-based catalysts for molecular O_2_ activation. The complementary characterization techniques and the first-principles calculations reveal that nitrogen vacancies not only possesses extraordinary capability to stabilize the metallic Pt nanoparticles, but also assists to donate electrons to the adsorbed O_2_. In catalytic toluene oxidation, the catalyst with Pt loading as low as 0.3 wt% exhibits excellent catalytic performance, which could completely convert toluene (1000 ppm) to CO_2_ at about 190 °C (T_100_). This conversion temperature is about 30 °C lower than that of the well-known Pt/CeO_2_ with the same amount of Pt loading, and the catalyst effectively reduce the activation energy of this reaction. In addition, the catalyst exhibits water-resistant properties and unexpected stability. Kept in air for 1 year as well as after hydrothermal aging treatment, the catalyst maintained the high activity. This work demonstrated an alternative concept to develop highly active and stable Pt-based oxidation catalysts. The prototype non-oxide support exemplified here could be extended to prepare other efficient metal-based catalysts.

## Results

### Identifying the formation of nitrogen vacancies

**Fig. 1:**
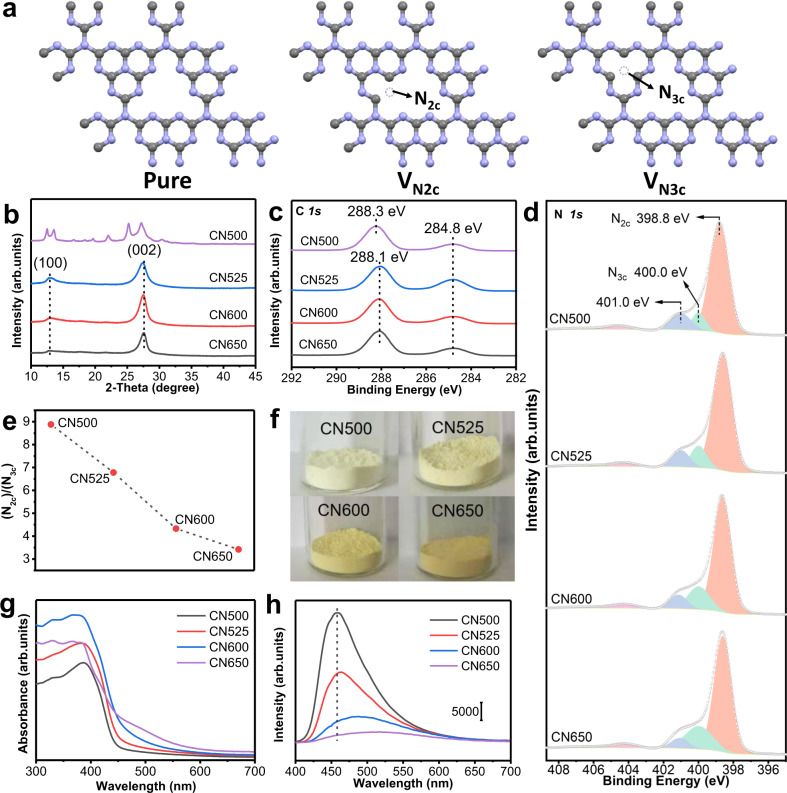
Structure, surface, and optical properties of different carbon nitride supports. **a** Schematic structures of the carbon nitrides with and without nitrogen vacancies. (gray represents C, purple represents N). **b**–**h** Various characterizations: **b** X-ray diffraction (XRD) patterns. **c** C 1*s* X-ray photoelectron spectra (XPS). **d** N 1*s* XPS spectra. **e** The (N_2c_)/(N_3c_) ratio calculated by the high-resolution N 1*s* XPS spectra. **f** Photographs of different samples. **g** UV–Vis diffuse reflectance spectra. **h** Photoluminescence (PL) emission spectra, excitation wavelength: 380 nm.

Nitrogen vacancies could be engineered by controlling the polymerization degrees of carbon nitride. We selected synthetic conditions that favor the higher condensation of melamine into corresponding products (temperatures between 500 and 650 °C), aiming to meet the stability requirement of application in vapor-phase reactions. The resultant samples are denoted as CN500, CN525, CN600, and CN650 according to the preparation temperatures. Two possible vacancy sites including vacancy of N_2c_ and N_3c_ are shown and denoted as V_N2c_ and V_N3c_, respectively, in Fig. 1a. According to our thermodynamic calculation, the formation of the neutral defects V_N2c_ and V_N3c_ are equally possible due to the slight energy difference of 0.025 eV/site. According to the bader charge analysis, the extra electrons introduced by V_N2c_ or V_N3c_ are not localized on the dangling carbon atoms, but re-distributed on the adjacent carbon atoms via the π-conjugated networks of polymeric carbon nitride.

The XRD patterns show that CN500 is an oligomer sample containing a melem related structure (Fig. 1b)^27–30^. The samples of CN525, CN600, and CN650 possess typical melon structure^27,31,32^. The structure change is due to the condensation of framework at higher temperature. That directly influence interplanar stacking distance, long-range order of the in-plane structural packing, specific surface area and surface defects (Supplementary Table 1, Supplementary Figs. 1 and 2).

X-ray photoelectron spectroscopy (XPS) was carried out to detect the atomic structure changes on the surface of these samples. The C 1*s* peak at 288.1 eV can be assigned to a C(N)_3_ coordination^31–33^, accompanied with a signal from adventitious carbon at 284.8 eV (Fig. 1c). The sharp N 1*s* XPS signal with a shoulder (Fig. 1d) can be fitted into peaks assigned to C–N–H (401.0 eV), N-(C)_3_ (400.0 eV), and C–N=C (398.8 eV) (Supplementary Table 2)^32,34–36^. Both the shift of N 1*s* XPS signal and changes of the ratio between C–N=C (denoted as N_2C_, see in Fig. 1a) and N-(C)_3_ (denoted as N_3C_, see in Fig. 1a) can be observed from the spectra. The N_2C_/N_3C_ ratio drops drastically from 8.88 of CN500 to 3.42 of CN650 (Fig. 1e). Correspondingly, the surface C/N atomic ratio increase from 0.65 to 0.73 (Supplementary Table 3). These experimental results indicate that surface nitrogen vacancies are formed during the condensation at high temperature and are mainly located at the N_2c_ lattice sites. In agreement with the theoretical expectation, extra electrons are found on some of the carbon atoms, which is further confirmed by the shift of C 1*s* peak to the low binding energy (Fig. 1c).

The optical characters further verify the formation of nitrogen vacancies in the samples prepared at high temperature. The color changes from pale yellow (CN500, Fig. 1f) to light brown (CN650). In agreement with the observed color, a gradual red-shifted absorption threshold can be observed in the samples of CN525, CN600, and CN650 (Fig. 1g). This red-shifted absorption threshold is originated from the vacancy-induced delocalization of the π-electron in the conjugated system^37^. The gradual enhancement of this signal indicates the larger amount of nitrogen vacancies can be created at a higher preparation temperature. The photoluminescence (PL) spectra (Fig. 1h and Supplementary Fig. 3) show that CN500 exhibits a strong emission peak at 458 nm. This emission is mainly originated from the band-to-band recombination electrons and holes. With the increasing preparation temperatures, the intensity of the emission peak obviously decreases. The time-resolved PL spectra (Supplementary Fig. 4 and Supplementary Table 4) show that the lifetimes of charges in these samples also exhibit a decrease trend. Combined these results, it is obtained that nonradiative recombination increases in the samples prepared at high temperatures, and the formation of nitrogen vacancies occurs during the condensation at high temperature.

### Anchoring Pt nanoparticles on carbon nitride supports

**Fig. 2:**
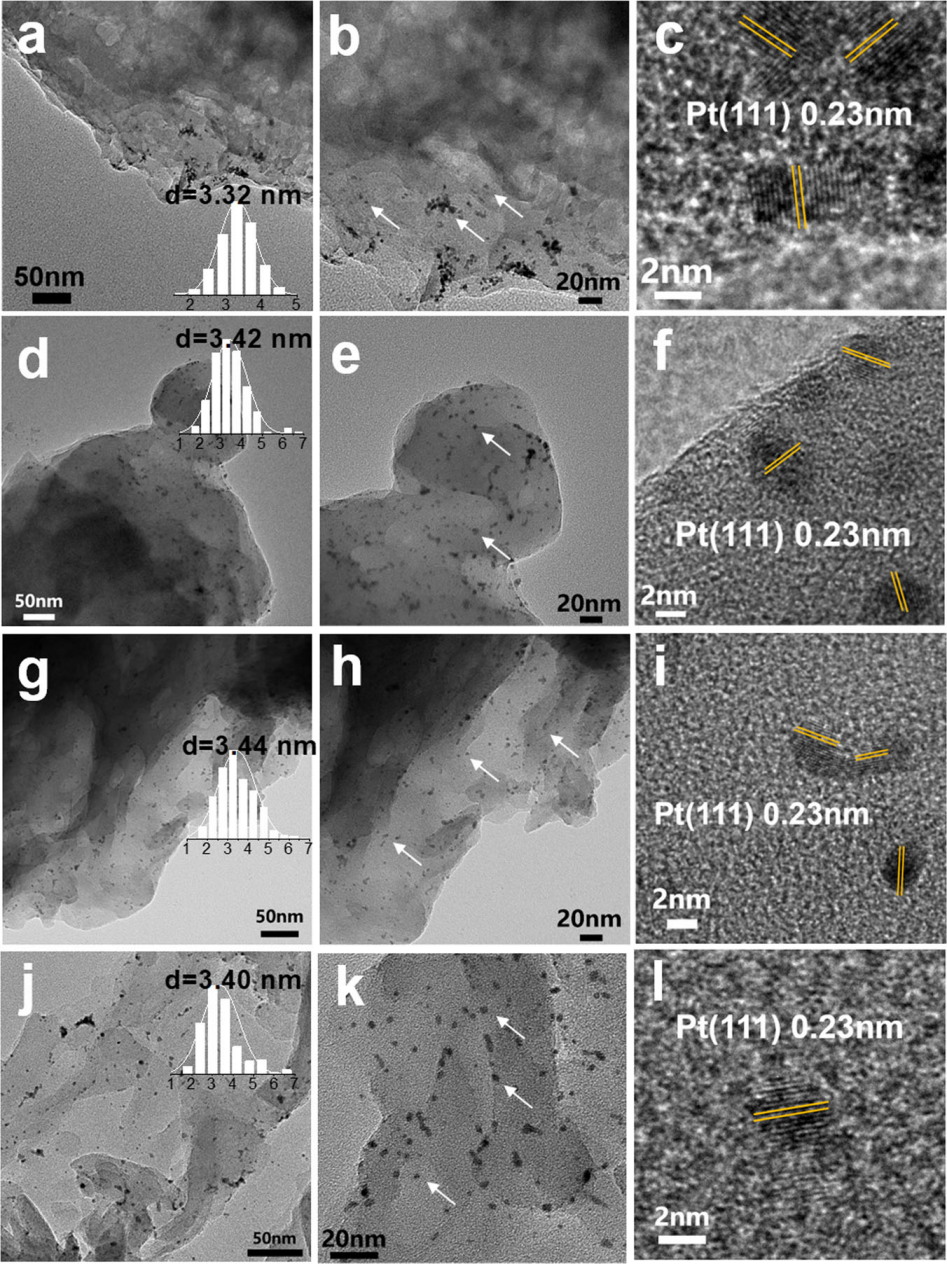
TEM and HRTEM images of different supported Pt samples. **a**–**c** Pt/CN500. **d**–**f** Pt/CN525. **g**–**i** Pt/CN600. **j**–**l** Pt/CN650.

Pt nanoparticles prepared by a polyol reduction method were used as a precursor to synthesize the catalysts^8,38^. The size of Pt nanoparticles were 3–4 nm in the colloid (Supplementary Fig. 5). In typical catalysts, the loading amount of Pt was controlled at 0.3 wt%. These Pt nanoparticles exhibit different dispersion behaviors over the carbon nitrides prepared at different temperatures (Fig. 2). Both isolated and aggregated behaviors of Pt nanoparticles can be observed over CN500 support (Fig. 2a, b). No Pt diffraction signals can be observed in the XRD patterns (Supplementary Fig. 6), indicating that the aggregated Pt nanoparticles maintain isolated and has not grown into large particles. As for the other three samples (CN525, CN600, and CN650), only isolated Pt nanoparticles (Fig. 2, marked by arrow, and Supplementary Fig. 7) can be observed. The statistical results show that these nanoparticles preserve the size of Pt nanoparticles in the colloid. The lattice fringe with a *d* spacing of 0.23 nm was observed in the HRTEM images, which could be ascribed to the (111) plane of Pt nanoparticles. During the loading process, the structural and surface properties of carbon nitrides are almost not affected, which are ascertained by various measurements including XRD, FT-IR, SEM, XPS, UV–Vis, and PL spectra (Supplementary Figs. 6, 8–16, Supplementary Table 5 and 6).

**Fig. 3:**
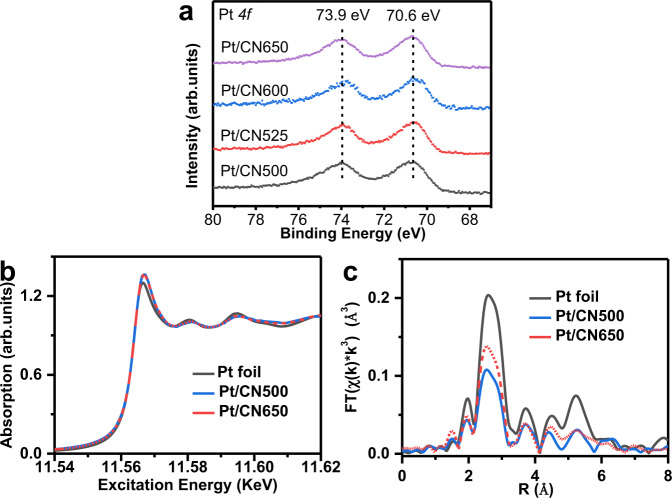
Chemical states of Pt nanoparticles over different carbon nitride supports. **a** Pt 4*f* XPS spectra. **b**, **c** X-ray absorption spectra. **b** X-ray absorption near edge structure (XANES) region. **c** The k^3^-weighted FT-EXAFS results.

Given the above characterization results, it can be concluded that the presence of nitrogen vacancies facilitates the dispersion of Pt on the surface of carbon nitride. This deduction could be further confirmed by our calculation result. According to the first-principles calculation, Pt atom can easily passivate the left dangling carbon atoms around V_N2c_ by gaining −2.33 eV rather than V_N3c_ by costing 0.43 eV. This result represents that V_N2c_ should be responsible for anchoring Pt. This distinguished binding energy might be ascribed to the large space around V_N2c_, which allows the p-d coupling of the Pt and the neighbored C and N atoms. More interestingly, merely slight charge transfer (about 0.23 e) is found between the Pt atom and the bonded atoms, which suggests the passivated Pt is more like a metal. The chemical states of Pt nanoparticles were analyzed using Pt 4*f* XPS spectra (Fig. 3a). All these four samples exhibit similar Pt 4*f* peaks, centering at 70.6 and 73.9 eV. Comparing with the oxides-supported-Pt catalysts, the binding energy obviously shift to a low value, indicating that most of Pt nanoparticles maintain the metallic state^13,16,18^. Synchrotron X-ray absorption experiments were also performed to further investigate the oxidation states of the Pt nanoparticles. In the X-ray absorption near edge structure (XANES) region, the edge energy of the samples of Pt/CN500 and Pt/CN650 are similar to that of Pt foil (Fig. 3b), indicating the metallic character. The extended X-ray absorption fine structure (EXAFS) spectra of these samples in R-space were also compared (Fig. 3c). The EXAFS spectra of both samples contain a major peak corresponding to Pt–Pt, present at approximately 2.5 Å. The EXAFS spectra lacked a major peak representing a Pt–O bond, which would be located around 1.5 Å. This qualitative analysis helps to confirm the metallic state of Pt nanoparticles.

### Catalytic toluene oxidation over platinum–carbon nitride catalysts

**Fig. 4:**
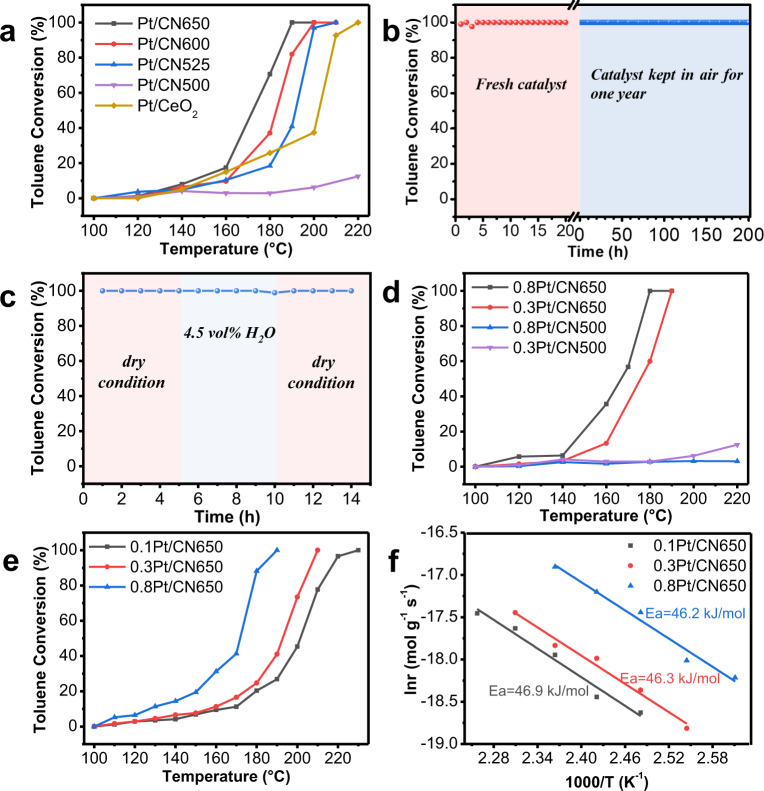
Catalytic activities of toluene oxidation over different supported Pt catalysts. **a** Toluene conversion as a function of temperature, Pt loading amount 0.3 wt%, reaction conditions: C_7_H_8_ 1000 ppm, space velocity (SV) = 24,000 mL g^−1^ h^−1^, air balance. **b** Stability test of Pt/CN650 for toluene oxidation, reaction temperature: 190 °C, the other reaction conditions are the same as **a**. **c** Influence of gas-phase water on toluene oxidation over Pt/CN650, H_2_O 4.5 vol%, the other reaction conditions are the same as **b**. **d** Toluene conversion over Pt/CN650 and Pt/CN500 with different loading amount of Pt, reaction conditions are the same as **a**. **e** Toluene conversion as a function of temperature over Pt/CN650 with different loading amount of Pt at SV of 60,000 mL g^−1^ h^−1^, the other reaction conditions are the same as **a**. **f** Arrhenius plots for toluene oxidation over Pt/CN650 with different loading amount of Pt.

Catalytic toluene oxidation was carried out to detect the catalytic performance of these catalysts (Fig. 4). In absence of catalysts, no toluene conversion is observed in the test temperature region. Figure 4a shows that the toluene catalytic conversions over Pt/CN525, Pt/CN600, and Pt/CN650 exhibit S-shaped curves. The toluene conversion increases upon the increase of reaction temperature. Among these three samples, Pt/CN650 exhibits the highest activity and nearly all toluene is selectively converted to CO_2_. *T*_50_ (the reaction temperature of toluene with 50% conversion) and *T*_100_ (complete conversion temperature of toluene) were achieved at 173 °C, 190 °C for Pt/CN650. The reaction temperature is about 30 °C lower than that of Pt/CeO_2_ with the same amount of Pt loading (Fig. 4a). CeO_2_ is a well-known redox metal-oxide support and has been recognized as a key component in the three-way catalytic converters for O_2_ activation^39,40^. In our case, CN650 obviously has advantages over CeO_2_. Besides, the performance of Pt/CN650 is even comparable with the samples with high Pt-loading (Supplementary Table 7).

It should be noted that Pt/CN650 also exhibits high stability in the reaction process. Even after storage in air for 1 year, there is almost no change for the conversion of toluene in the test period at 190 °C (Fig. 4b). Moreover, water gas in the reactants has no effect on the catalytic performance of Pt/CN650 (Fig. 4c). This is a desirable property for the catalysts used in emissions control since water vapor inevitably exists in exhaust emission. It was reported that many catalysts would deactivate in a certain degree in the presence of water gas^4^. Besides, we have also tested the catalytic performance of Pt/CN650 after hydrothermal aging treatment. There is almost no difference between fresh and treated samples (Supplementary Fig. 17). It should be noted that all above catalytic results are obtained at the low-Pt-contents of 0.3 wt%. Considering the cost of the catalysts and the energy consuming of the reaction, it takes a great advantage to achieve the complete conversion of toluene at such a low temperature and low amount of Pt loading.

**Fig. 5:**
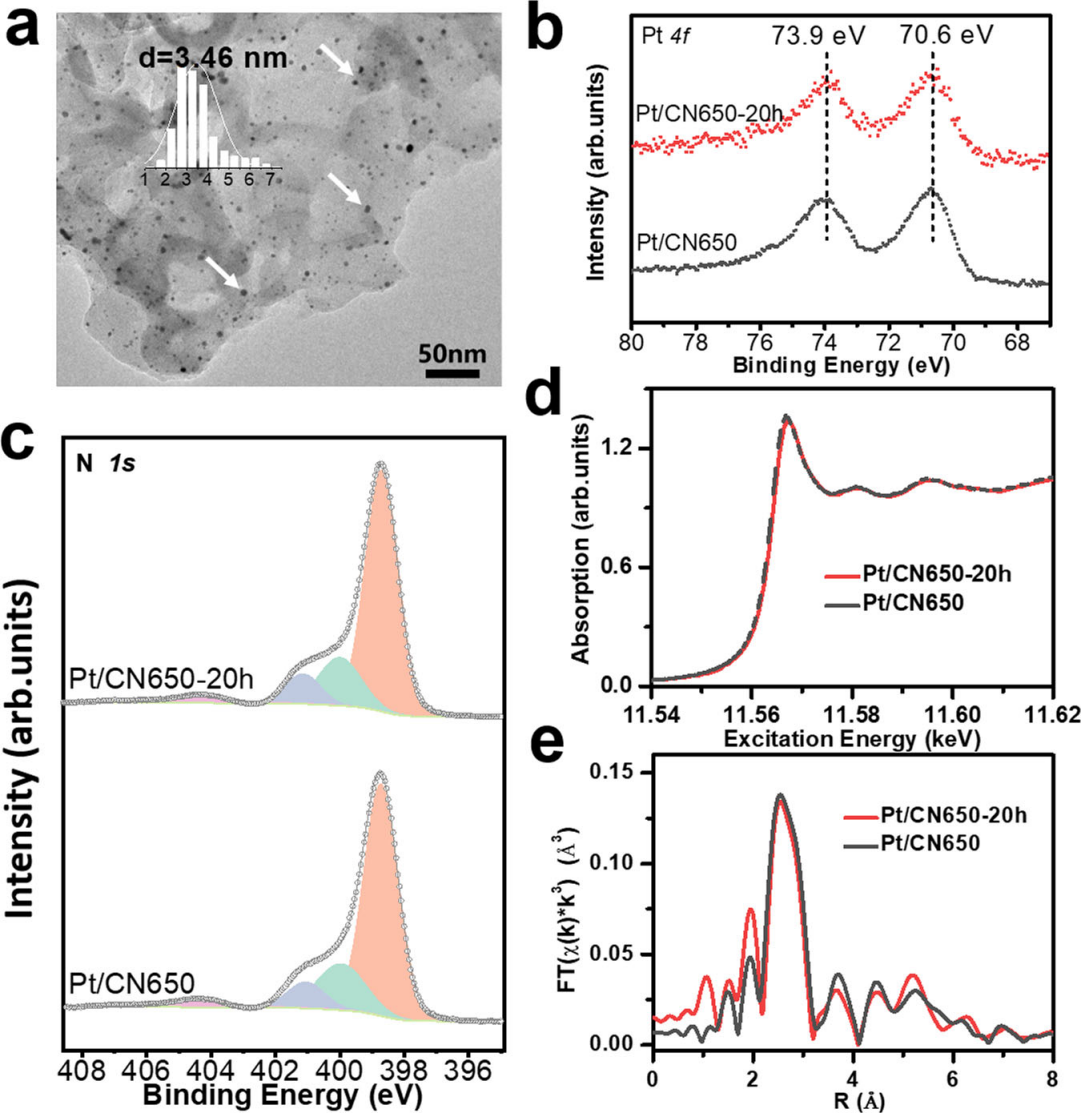
Structural and surface properties of fresh and used Pt/CN650. **a** TEM image. **b** Pt 4*f* XPS spectra. **c** N 1*s* XPS spectra. **d** XANES. **e** EXAFS.

The stability of catalytic active sites in the reaction process is a critical factor for practical application. For this catalyst, it includes the changes of the morphology and the chemical states of the Pt nanoparticles and the carbon nitride support. In this work, we systematically compared the properties of fresh Pt/CN650 and the one used for 20 h reaction (denoted as Pt/CN650-20h). The SEM, TEM images, and XRD patterns show that Pt/CN650-20h maintains the morphology and structure of Pt/CN650 (Fig. 5a, Supplementary Figs. 18,  and  19). Pt nanoparticles are highly dispersed on the surface of the carbon nitride support and no aggregation is observed in the detect regions (Fig. 5a). As shown in the Pt 4*f* XPS, Pt maintains in metallic states after long-time reaction (Fig. 5b), which is ascertained by the XANES (Fig. 5d) and EXAFS measurements (Fig. 5e). More importantly, N 1*s* and C 1*s* results also show the well-maintained surface chemical states of carbon nitride support (Fig. 5c and Supplementary Fig. 20). The ratio between C–N–H, N-(C)_3_, and C–N=C is almost the same before and after the reaction (Supplementary Table 8). All above results show that the nitrogen vacancies and metallic Pt are both stable during the catalytic toluene oxidation.

Considering the effect of loading amount, we further investigated the catalytic oxidation of toluene over the catalysts with higher Pt loading (0.8 wt%). Under the same reaction conditions as 0.3 wt% Pt/CN650 (space velocity, SV = 24,000 mL g^−1^ h^−1^), *T*_50_ and *T*_100_ can be achieved at 166 °C, 180 °C over 0.8 wt% Pt/CN650 (Fig. 4d). Interestingly, these Pt/CN650 samples also exhibit high activity under high SV (60,000 mL g^−1^ h^−1^). Toluene could be completely converted to CO_2_ at about 190 °C over 0.8 wt% Pt/CN650 under such high SV (Fig. 4e). The apparent activation energies are estimated by using the Arrhenius relationship (Fig. 4f). The activation energy over Pt/CN650 with different Pt contents is 46.2 kJ mol^−1^ (0.8 wt%Pt/CN650), 46.3 kJ mol^−1^ (0.3 wt%Pt/CN650) and 46.9 kJ mol^−1^ (0.1 wt%Pt/CN650), respectively. To our knowledge, this value is much lower than that of the most reported oxides-supported catalysts including the optimized Pt/Al_2_O_3_ catalyst recently reported by our group^8,41–44^. Besides, these Pt/CN catalysts show very similar apparent activation energy, which indicates that the increase of Pt loading amount mainly improves the numbers of active sites for catalytic oxidation.

In comparison, over 0.3 wt% Pt/CN500, only 12.3% toluene conversion can be observed when the reaction temperature is up to 220 °C (Fig. 4a). Increasing the amount of Pt to 0.8 wt% over CN500 has no significant impact on the catalytic activity. (Fig. 4d). One possible reason is the aggregation of Pt nanoparticles over CN500 support. However, it is insufficient to explain the low activity of Pt/CN500 since a certain amount of isolated Pt nanoparticles can be observed on the surface of CN500 (Fig. 2b). The morphology of the Pt is similar to that of the sample Pt/CN650 (Fig. 2k). Both the XPS and the X-ray absorption results show that Pt nanoparticles possess similar metallic properties in the samples of Pt/CN500 and Pt/CN650 (Fig. 3 and Supplementary Fig. 21). We also detected the catalytic performance of CO oxidation on these catalysts. It exhibits similar activity trend to that in toluene oxidation (Supplementary Fig. 22), which is 0.8 wt%Pt/CN650 > 0.3 wt%Pt/CN650 » 0.8 wt%Pt/CN500 ≈ 0.3 wt%Pt/CN500. Undoubtedly, the metallic Pt nanoparticles serve as the active sites for CO adsorption. The different catalytic activities over different carbon nitride supports further implies the participation of the nitrogen vacancies in the reaction process.

Diffuse-reflectance infrared Fourier transform (DRIFT) analysis of toluene adsorbed onto the catalyst surface, and temperature-programmed desorption of toluene (C_7_H_8_-TPD) were carried out to investigate the performance of catalysts in the activation/adsorption of toluene (Supplementary Figs. 23 and  24). DRIFT spectra of toluene adsorption show that no obvious difference between two typical samples, Pt/CN500 and Pt/CN650 (Supplementary Fig. 23). In the profiles of C_7_H_8_-TPD, the C_7_H_8_ desorption area over Pt/CN500 is larger than that of Pt/CN650 (Supplementary Fig. 24). According to these two results, it can be ruled out that Pt/CN650 has an advantage on the activation of toluene in comparison with Pt/CN500. The influence of PVP could also be excluded. Similar reaction results could be obtained in the absence of PVP during preparation (Supplementary Fig. 25). Considering the defect-state of carbon nitride supports, the participation of nitrogen vacancies in molecular O_2_ activation should be responsible for the catalytic activities.

**Fig. 6:**
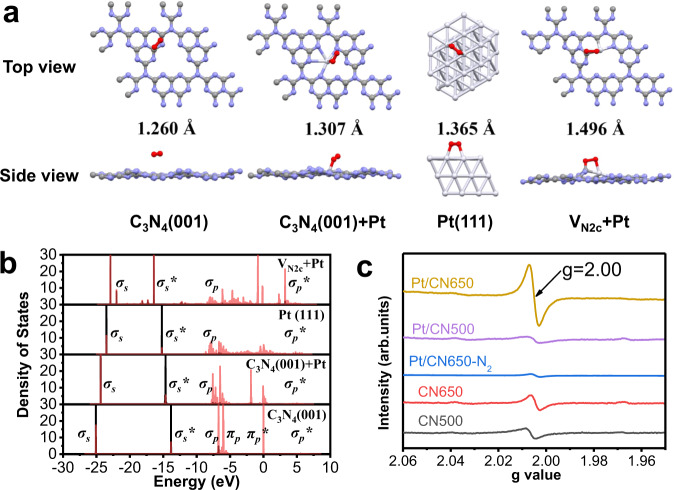
Mechanistic insights of catalytic toluene oxidation over the catalysts. **a** The structures of O_2_ adsorbed on pure C_3_N_4_ (001), C_3_N_4_ (001) +Pt, Pt (111), and V_N2c_ + Pt (gray, purple, white, and red represents C, N, Pt, and O). **b** Partial density of states (PDOS) of the adsorbed O_2_ (The black and red lines represent the O 2*s* and 2*p* orbitals, respectively. The molecular orbitals are also denoted). **c** EPR spectra of carbon nitrides and supported Pt catalysts at room temperature in air and N_2_ atmosphere.

The adsorption and activation of O_2_ on the catalyst is recognized as the key step of the whole reaction. To understand the mechanism of the enhanced O_2_ activation in this system, we have performed the first-principles calculation of the O_2_ adsorption on C_3_N_4_(001), Pt-adsorbed C_3_N_4_ (001) (C_3_N_4_(001)+Pt), Pt (111), and Pt passivated nitrogen vacancy (V_N2c_ + Pt), respectively, as shown in Fig. 6a. The O–O bond length of the adsorbed O_2_ molecule is 1.260, 1.307, 1.365, and 1.496 Å, respectively, on different substrates. The gradual elongated bond-lengths indicate the gradual enhanced activation of O_2_ due to the electron donation of the substrates. Particularly when the O_2_ is adsorbed on V_N2c_ + Pt, it is found that about 0.65 electron transferred from the passivated Pt and the dangling carbon atoms to the O_2_ molecule, which occupies the antibonding state (*σ*_p_*) as shown in Fig. 6b top. Adsorbed solely on the Pt (111), Pt-adsorbed C_3_N_4_ (001), or C_3_N_4_ (001) surface, the charge transfer is about 0.39, 0.21 electron or nearly zero, respectively. With electron further occupying the *σ*_p_^*^ state, the *σ*_p_ bond of O_2_ molecule is expected to break. The weak interaction of the bonding state (*σ*_s_) and antibonding state (*σ*_s*_) of the adsorbed O_2_ on Pt + V_N2c_ also suggests the elongation of the O–O bond, which facilitates the further reaction of O–O cleavage. Comparing the non-defective and defective cases, the contribution of nitrogen-vacancy should be the main reason to reduce the activation energy of the catalytic toluene oxidation.

In experiment, electron paramagnetic resonance (EPR) was carried out to investigate the capability of Pt/CN500 and Pt/CN650 for the activation of molecular oxygen. Exposed to air at room temperature, Pt/CN650 exhibit an obvious signal with a *g* factor of 2.00 (Fig. 6c). This signal can be ascribed to surface superoxide radical anion O_2_^**·-**^ species. As for Pt/CN500, this signal is very weak. We also measure the EPR properties of Pt/CN650 under the nitrogen atmosphere. Only a weak signal can be observed in the absence of molecular oxygen. This result confirms that Pt/CN650 possesses excellent capability to activate molecular oxygen at low temperatures.

Very weak EPR signals were detected over the pure CN500 and CN650 supports. This means the unpaired electrons of carbon nitride support alone cannot effectively initiate the activation of molecular oxygen at low temperatures. Comparing the EPR signal for Pt/CN500 and Pt/CN650, the signal for Pt/CN500 is quite weak, although the Pt nanoparticles in the two samples are both in metallic states. This difference suggests that metallic Pt nanoparticles cannot independently undertake the responsibility of molecular oxygen activation at low temperatures either. Otherwise, the synergism of metallic Pt nanoparticles and nitrogen vacancies of carbon nitride support should be responsible for the high performance of O_2_ activation at low temperatures.

## Discussion

In summary, nitrogen vacancies of carbon nitride significantly enhance the O_2_ activation of Pt-based catalysts. The metallic Pt are well maintained over the manipulated carbon nitride. The Pt atoms and the dangling carbon atoms surround the vacancies synergistically donate electrons to the antibonding orbital of the adsorbed O_2_, effectively rendering O_2_ more reactive toward reactants and lowering the activation energy of the reaction. This work provides a facile manufacture way to fabricate highly efficient supported-Pt catalyst for catalytic oxidation. It should be noted that this strategy is of great potential to further enhance the activity. The small specific surface area of carbon nitride used in this work obviously limit the adsorption of toluene. Rapid progress in the synthesis of non-oxide materials would provide much opportunity. The adsorption and activation of pollutant molecules could be easily increased by tuning the texture properties of this non-oxide support. The concept of increased electron donors induced by nitrogen vacancy could be extended to develop a series of supported metal nanoparticles catalysts with diverse functionalities.

## Methods

### Materials

Melamine and polyvinyl pyrrolidone (PVP) were purchased from Tianjin Guangfu Fine Chemical Research Institute. H_2_PtCl_6_•6H_2_O was purchased from Sinopharm Chemical Reagent Co., Ltd. NaOH, HCl, and ethylene glycol were obtained from Beijing Chemical Works. All chemical reagents were analytical grade and without purification before used.

### Preparation of different carbon nitride supports

Different carbon nitride samples were prepared by heat treatment of melamine precursors. Five gram of melamine was sealed in a ceramic crucible and placed into muffle furnace. It was heated up to corresponding temperatures (500, 525, 600, and 650 °C) with a heating rate of 10 °C min^−1^, and kept at this temperature for 2 h. The product was cooled down to room temperature and ground to powder. The obtained samples were denoted as CN500, CN525, CN600, and CN650, respectively.

### Preparation of Pt nanoparticles

Pt nanoparticles were prepared by a polyol reduction method which was reported in our previous work. In a typical case, 15 mL NaOH-glycol solution (0.5 mol L^−1^) was added into 27 mL H_2_PtCl_6_·6H_2_O-glycol solution (11.4 mmol L^−1^). The pale-yellow transparent solution was heated at 90 °C for 2 h under N_2_ atmosphere. After cooling to room temperature, HCl (0.77 mol L^−1^) was dropwise added into the colloid until the pH value was adjusted to 3.0. The Pt nanoparticles were collected by centrifugation and subsequently dispersed in PVP-aqueous solution.

### Preparation of different Pt/CN catalysts

The catalysts were prepared by an impregnation method. Typically, a certain amount of carbon nitride powder was added into the Pt PVP-aqueous solution and the mixture was stirred for 0.5 h, and subsequently evaporated to remove the water at 80 °C. The stoichiometrically content Pt was controlled to 0.3 wt%. The obtained samples were calcined at 250 °C in 20 vol%O_2_/Ar flow for 2 h and the resultant powder was named as Pt/CN500, Pt/CN525, Pt/CN600, and Pt/CN650.

### Characterization

X-ray diffraction (XRD) patterns were performed on an Empyrean X-ray diffractometer with Cu K_α_ radiation source. FT-IR measurements were operated on a Nicolet 6700 spectrometer. N_2_-adsorption/desorption tests were carried out on the ASAP 2010N instrument. The surface areas were calculated based on the model of Brunauer–Emmett–Teller (BET). X-ray photoelectron spectroscopy (XPS) were recorded on a Thermo ESCA LAB 250 instrument. The high-resolution transmission electron microscopy (HRTEM) images were collected on a JEM-2100F instrument. Electron paramagnetic resonance (EPR) results were obtained on a JES-FA 200 EPR spectrometer. The UV–Vis diffuse reflectance spectra were measured on a Shimadzu-3600 UV–Vis–NIR spectrophotometer. The photoluminescence (PL) spectra were carried out on the FLS920 (Edinburgh Instrument) at room temperature and the excitation wavelengths were 380 and 365 nm. The emission decay lifetime was measured at 485 nm.

XAS measurements were performed using the Sector 9-BM beamline of the Advanced Photon Source at Argonne National Laboratory (Argonne, IL). The end station was equipped with a double-crystal Si (111) monochromator for wavelength selection. A 12-element Ge fluorescence detector was used to collect spectra. Sample powders were packed on plastic washers to enhance the signal. Data processing was performed using WinXAS software.

DRIFT spectrum was collected at 140 °C in 20 vol% O_2_/Ar atmosphere. The sample was pretreated at 200 °C in N_2_ for 15 min before testing. C_7_H_8_-TPD was obtained by subtracting the blank spectrum of the samples and the procedure were as follows. Firstly, 50 mg of the samples were pretreated at 200 °C in Ar flow for 30 min. After cooling to 70 °C, toluene was introduced for adsorption by gas bubbling method, the time for adsorption was 30 min. Then treating the samples were maintained at 70 °C in Ar flow for 1 h, and collected the signal with temperature rising. The blank spectrum of the sample is obtained in the same way except for the toluene adsorption.

## Method and models for the first-principles calculations

All the first-principles calculations in this work are performed by the VASP code^45,46^. The PAW pseudopotentials and optPBE-vdw functional are employed for structural optimization with an energy cutoff of 520 eV^47,48^. The 2 × 2 × 1 slab models containing respective three atomic layers are adopted with the vacuum layer of 20 Å for C_3_N_4_ (001) and Pt (111). The bottom two layers and the lattice vectors are fixed, and the top one layer and the adsorbed atoms are fully relaxed with 2 × 2 × 1 and 5 × 5 × 1 Monkhorst-Pack k-point sampling until the force on each atom is less than 0.01 eV/Å for the C_3_N_4_ (001) and Pt (111) facet, respectively. Although complicated models with multiple N-vacancies and Pt atoms cannot be ruled out, simple cases including single type of defects and single Pt atom are found enough to describe the synergistic effect of the support and Pt in this work.

### Catalytic performance evaluation

The catalytic activity tests were performed at a continuous-flow fix-bed reactor. For toluene oxidation, 100 mg of the catalyst with 40–60 mesh were placed into the central position of the quartz reactor. Toluene was introduced into the reactor by bubbling air stream through a toluene reservoir (keep in an ice bath). Another air stream was used to achieve toluene vapor concentration of 1000 ppm. The concentrations of reactants and products are analyzed by an online gas chromatograph equipped with an FID detector for toluene and a TCD detector for CO_2_.

Toluene conversion (*X*_tol_) is calculated as Eq. (1):

1 $${X}_{{{\rm{tol}}}}\,=\,\frac{{C}_{{{\rm{in}}}}-{C}_{{{\rm{out}}}}}{{C}_{{{\rm{in}}}}}\times 100 \%$$

Specific reaction rates for toluene oxidation at different temperatures are evaluated under the condition of toluene conversion is below 15%. For each run at a specific temperature, toluene conversions are averaged at the steady state and the specific rates are calculated as Eq. (2):

2 $${r}_{{{\rm{tol}}}}\,=\,\frac{{X}_{{{\rm{tol}}}}{F}_{{{\rm{tol}}}}}{{m}_{{{\rm{cat}}}}},$$

where *X*_tol_ is the toluene conversion at different temperatures, *F*_tol_ is the molar flow rate of toluene and *m*_cat_ is the mass of catalyst.

In the condition of excess oxygen, toluene oxidation has been reported following first-order kinetics to toluene concentration^49^, which follows Eq. (3):

3 $$r=-{kc}$$

The apparent activation energies are calculated based on the Arrhenius Eq. (4):

4 $${{\rm{ln}}k}\,=\,-\frac{{E}_{{\rm{a}}}}{{RT}}\,+\,{{\rm{ln}}}A,$$

where *k* is the rate constant, *E*_a_ is the apparent activation energy, and *A* is the pre-exponential factor.

As for CO oxidation, 80 mg of the catalyst with 40–60 mesh are used. The inlet reactant gas is 1 vol% CO, 5 vol% O_2_, and Ar balanced. The total gas flow rate is 100 mL min^−1^ and the space velocity is 75,000 mL g^−1^ h^−1^. The outlet reactants and products are analyzed by an online gas chromatograph equipped with a TCD detector. CO conversion (*X*_CO_) is calculated as the formula (5):

5 $${X}_{{{\rm{CO}}}}\,=\,\frac{{C}_{{{\rm{in}}}}-{C}_{{{\rm{out}}}}}{{C}_{{{\rm{in}}}}}\times 100 \%$$

## Supplementary information

Supplementary Information

Peer Review File

## Data Availability

The data that support the findings of this study are available within the article and its Supplementary Information files. All other relevant data supporting the findings of this study are available from the corresponding authors upon request. Source data are provided with this paper.

## Source data

Source Data

## References

[1] Pu T, Tian H, Ford ME, Rangarajan S, Wachs IE (2019). Overview of selective oxidation of ethylene to ethylene oxide by Ag catalysts. ACS Catal..

[2] Montemore MM, van Spronsen MA, Madix RJ, Friend CM (2018). O_2_ activation by metal surfaces: implications for bonding and reactivity on heterogeneous catalysts. Chem. Rev..

[3] He C (2019). Recent advances in the catalytic oxidation of volatile organic compounds: a review based on pollutant sorts and sources. Chem. Rev..

[4] Liotta LF (2010). Catalytic oxidation of volatile organic compounds on supported noble metals. Appl. Catal. B.

[5] Kamal MS, Razzak SA, Hossain MM (2016). Catalytic oxidation of volatile organic compounds (VOCs)—a review. Atmos. Environ..

[6] Liu G, Walsh AG, Zhang P (2020). Synergism of iron and platinum species for low-temperature CO oxidation: from two-dimensional surface to nanoparticle and single-atom catalysts. J. Phys. Chem. Lett..

[7] Hoang S (2019). High performance diesel oxidation catalysts using ultra-low Pt loading on titania nanowire array integrated cordierite honeycombs. Catal. Today.

[8] Gan T (2019). Pt/Al_2_O_3_ with ultralow Pt-loading catalyze toluene oxidation: promotional synergistic effect of Pt nanoparticles and Al_2_O_3_ support. Appl. Catal. B.

[9] Yashnik SA, Ismagilov ZR, Porsin AV, Denisov SP, Danchenko NM (2007). Development of monolithic catalysts with low noble metal content for diesel vehicle emission control. Top. Catal..

[10] Ren J, Wang Y, Zhao J, Tan S, Petek H (2019). K atom promotion of O_2_ chemisorption on Au(111) surface. J. Am. Chem. Soc..

[11] Allian AD (2011). Chemisorption of CO and mechanism of CO oxidation on supported platinum nanoclusters. J. Am. Chem. Soc..

[12] Abednatanzi S (2020). Metal-free activation of molecular oxygen by covalent triazine frameworks for selective aerobic oxidation. Sci. Adv..

[13] Zheng B (2016). Room temperature CO oxidation over Pt/MgFe_2_O_4_: a stable inverse spinel oxide support for preparing highly efficient Pt catalyst. ACS Appl. Mater. Interfaces.

[14] Nie L (2017). Activation of surface lattice oxygen in single-atom Pt/CeO_2_ for low-temperature CO oxidation. Science.

[15] An K (2013). Enhanced CO oxidation rates at the interface of mesoporous oxides and Pt nanoparticles. J. Am. Chem. Soc..

[16] Hoang S (2020). Activating low-temperature diesel oxidation by single-atom Pt on TiO_2_ nanowire array. Nat. Commun..

[17] Zhang Z (2017). Thermally stable single atom Pt/m-Al_2_O_3_ for selective hydrogenation and CO oxidation. Nat. Commun..

[18] Jing P (2019). Synergism of Pt nanoparticles and iron oxide support for chemoselective hydrogenation of nitroarenes under mild conditions. Chin. J. Catal..

[19] Setvin M (2013). Reaction of O_2_ with subsurface oxygen vacancies on TiO_2_ anatase (101). Science.

[20] Vayssilov GN (2011). Support nanostructure boosts oxygen transfer to catalytically active platinum nanoparticles. Nat. Mater..

[21] Wang X (2009). A metal-free polymeric photocatalyst for hydrogen production from water under visible light. Nat. Mater..

[22] Wang X, Blechert S, Antonietti M (2012). Polymeric graphitic carbon nitride for heterogeneous photocatalysis. ACS Catal..

[23] Zhao Y (2014). Graphitic carbon nitride nanoribbons: graphene-assisted formation and synergic function for highly efficient hydrogen evolution. Angew. Chem. Int. Ed..

[24] Liu J, Wang H, Antonietti M (2016). Graphitic carbon nitride “reloaded”: emerging applications beyond (photo)catalysis. Chem. Soc. Rev..

[25] Ong WJ, Tan LL, Ng YH, Yong ST, Chai SP (2016). Graphitic carbon nitride (g-C_3_N_4_)-based photocatalysts for artificial photosynthesis and environmental remediation: Are we a step closer to achieving sustainability?. Chem. Rev..

[26] Zhu J, Xiao P, Li H, Carabineiro SA (2014). Graphitic carbon nitride: synthesis, properties, and applications in catalysis. ACS Appl. Mater. Interfaces.

[27] Lau VW (2015). Low-molecular-weight carbon nitrides for solar hydrogen evolution. J. Am. Chem. Soc..

[28] Kumar P (2019). C_3_N_5_: a low bandgap semiconductor containing an Azo-linked carbon nitride framework for photocatalytic, photovoltaic and adsorbent applications. J. Am. Chem. Soc..

[29] Jurgens B (2003). Melem (2,5,8-triamino-tri-s-triazine), an important intermediate during condensation of melamine rings to graphitic carbon nitride: synthesis, structure determination by X-ray powder diffractometry, solid-state NMR, and theoretical studies. J. Am. Chem. Soc..

[30] Zheng HB (2017). Melem: an efficient metal-free luminescent material. J. Mater. Chem. C.

[31] Singh JA, Overbury SH, Dudney NJ, Li M, Veith GM (2012). Gold nanoparticles supported on carbon Nitride: influence of surface hydroxyls on low temperature carbon monoxide oxidation. ACS Catal..

[32] Thomas A (2008). Graphitic carbon nitride materials: variation of structure and morphology and their use as metal-free catalysts. J. Mater. Chem..

[33] Niu P, Liu G, Cheng H-M (2012). Nitrogen vacancy-promoted photocatalytic activity of graphitic carbon nitride. J. Phys. Chem. C.

[34] Yang P, Ou H, Fang Y, Wang X (2017). A facile steam reforming strategy to delaminate layered carbon nitride semiconductors for photoredox catalysis. Angew. Chem. Int. Ed..

[35] Yu, Q., Lin, X., Li, X. & Chen, J. Photocatalytic stille cross-coupling on gold/g-C_3_N_4_ nano-heterojunction. *Chem. Res. Chin. Univ.* **36**, 1013–1016 (2020).

[36] Qi, Y., Xu, J., Wang, C., Zhan, T. & Wang, L. Synthesis of holey graphitic carbon nitride with highly enhanced photocatalytic reduction activity via melamine-cyanuric acid precursor route. *Chem. Res. Chin. Univ.* **36**, 1024–1031 (2020).

[37] Shi, L. et al. Photoassisted construction of holey defective g-C_3_N_4_ photocatalysts for efficient visible-light-driven H_2_O_2_ production. *Small* **14**, 1703142 (2018). 10.1002/smll.201703142 29319230

[38] Chen C (2015). Importance of platinum particle size for complete oxidation of toluene over Pt/ZSM-5 catalysts. Chem. Commun..

[39] Colussi S, Fornasiero P, Trovarelli A (2020). Structure-activity relationship in Pd/CeO_2_ methane oxidation catalysts. Chin. J. Catal..

[40] Wang L, Deo S, Dooley K, Janik MJ, Rioux RM (2020). Influence of metal nuclearity and physicochemical properties of ceria on the oxidation of carbon monoxide. Chin. J. Catal..

[41] Wang B (2018). Effects of dielectric barrier discharge plasma on the catalytic activity of Pt/CeO_2_ catalysts. Appl. Catal. B.

[42] Peng R (2016). Shape effect of Pt/CeO_2_ catalysts on the catalytic oxidation of toluene. Chem. Eng. J..

[43] Fu X (2020). Intermetallic compound PtMn_y_-derived Pt−MnO_x_ supported on mesoporous CeO_2_: Highly efficient catalysts for the combustion of toluene. Appl. Catal. A.

[44] Li S (2020). Polyhedral cobalt oxide supported Pt nanoparticles with enhanced performance for toluene catalytic oxidation. Chemosphere.

[45] Kresse G, Furthmüller J (1996). Efficient iterative schemes for ab initio total-energy calculations using a plane-wave basis set. Phys. Rev. B.

[46] Kresse G, Furthmüller J (1996). Efficiency of ab-initio total energy calculations for metals and semiconductors using a plane-wave basis set. Comput. Mater. Sci..

[47] Klimes J, Bowler DR, Michaelides A (2010). Chemical accuracy for the van der Waals density functional. J. Phys. Condens. Matter.

[48] Klimeš J, Bowler DR, Michaelides A (2011). Van der Waals density functionals applied to solids. Phys. Rev. B.

[49] Zhao S, Hu F, Li J (2016). Hierarchical core–shell Al_2_O_3_@Pd-CoAlO microspheres for low-temperature toluene combustion. ACS Catal..

