# Supplementary material for: Electron donation of non-oxide supports boosts O2 activation on nano-platinum catalysts
Source: Nat Commun. 2021 May 12;12:2741. doi: 10.1038/s41467-021-22946-y (PMC8115247; doi:10.1038/s41467-021-22946-y)
Supplement: Supplementary file 1 — Supplementary Information [file 41467_2021_22946_MOESM1_ESM.pdf]

Supplementary Information

Electron donation of non-oxide supports boosts O_2 activation on nano-Platinum catalysts

Gan *et al.*

Content

Supplementary Figures

Supplementary Figure 1| FT-IR spectra of carbon nitrides prepared at different temperatures.

Supplementary Figure 2| SEM images of carbon nitrides prepared at different temperatures.

Supplementary Figure 3| Photoluminescence (PL) emission spectra, excitation wavelength: 365nm.

Supplementary Figure 4| Time-resolved PL decay spectra of carbon nitrides prepared at different temperatures.

Supplementary Figure 5| TEM image of Pt nanoparticles in the colloid.

Supplementary Figure 6| XRD patterns of Pt supported on different carbon nitrides.

Supplementary Figure 7| HAADF-STEM image of 0.3wt% Pt/CN650.

Supplementary Figure 8| FT-IR spectra of Pt supported on different carbon nitrides.

Supplementary Figure 9| SEM images of Pt supported on different carbon nitrides.

Supplementary Figure 10| C *1s* spectra of Pt supported on different carbon nitrides.

Supplementary Figure 11| N *1s* spectra of Pt supported on different carbon nitrides.

Supplementary Figure 12| The (N2c)/(N3c) ratio calculated by the high-resolution N *1s* spectra.

Supplementary Figure 13| Photographs of Pt supported on different carbon nitrides.

Supplementary Figure 14| UV-vis diffuse reflectance spectra of Pt supported on different carbon nitrides.

Supplementary Figure 15| Photoluminescence (PL) emission spectra, excitation wavelength: a 380nm, b 365 nm.

Supplementary Figure 16| Time-resolved PL decay spectra of Pt supported on different carbon nitrides.

Supplementary Figure 17| Stability test of toluene oxidation over 0.3Pt/CN650 after different hydrothermal treatment.

Supplementary Figure 18| SEM image of Pt/CN650-20h.

Supplementary Figure 19| XRD patterns of Pt/CN650 and Pt/CN650-20h.

Supplementary Figure 20| C *1s* spectra of Pt/CN650 and Pt/CN650-20h.

Supplementary Figure 21| Pt *4f* spectra of 0.8wt% Pt/CN500 and 0.8wt% Pt/CN650.

Supplementary Figure 22| CO conversion as a function of temperature over Pt/CN650 and Pt/CN500 with different loading amount of Pt, reaction conditions: CO 1 vol%, O_2 5 vol%, SV=75000 $mL \cdot g^{-1} \cdot h^{-1}$, Ar balance.

Supplementary Figure 23| In-situ C_7H_8-adsorbed DRIFT spectra of Pt/CN500 and Pt/CN650.

Supplementary Figure 24| C_7H_8-TPD profiles of Pt/CN500 and Pt/CN650.

Supplementary Figure 25| Toluene conversion as a function of temperature over different samples.

Supplementary Tables

Supplementary Table 1| Texture properties of carbon nitrides prepared at different temperatures.

Supplementary Table 2| Surface functionalities calculated based on peaks fit to XPS spectra.

Supplementary Table 3| The ratio of C to N calculated based on the XPS results.

Supplementary Table 4| Emission decay lifetime of carbon nitrides prepared at different temperatures.

Supplementary Table 5| Surface functionalities calculated based on peaks fit to XPS spectra.

Supplementary Table 6| Emission decay lifetime of Pt supported on different carbon nitrides.

Supplementary Table 7| Catalytic activity for toluene oxidation over various catalysts

Supplementary Table 8| Surface functionalities based on peaks fit to XPS spectra.

Supplementary Figures

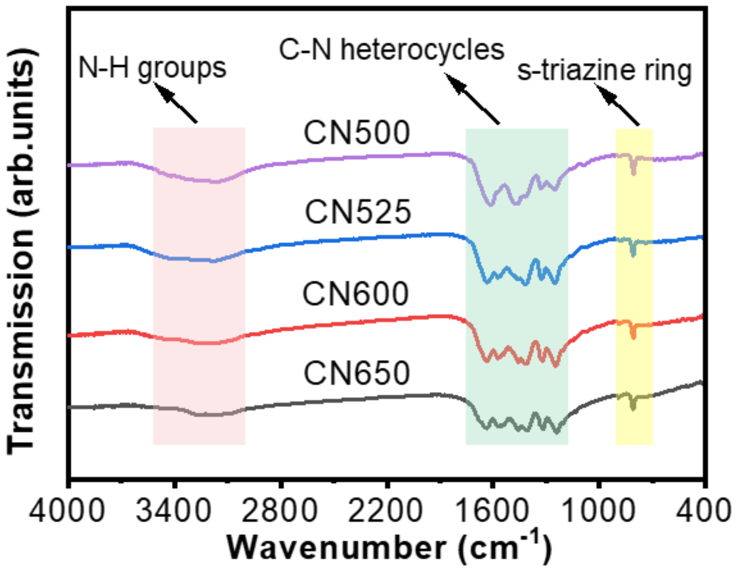

Supplementary Figure 1| FT-IR spectra of carbon nitrides prepared at different temperatures.

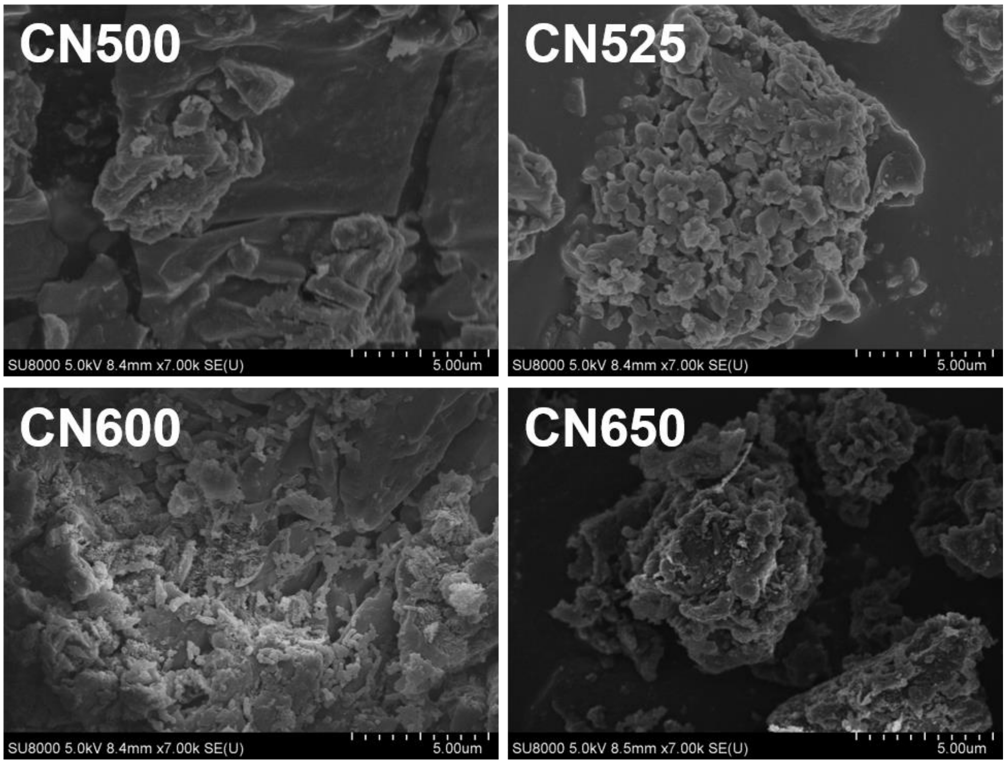

Supplementary Figure 2| SEM images of carbon nitrides prepared at different temperatures.

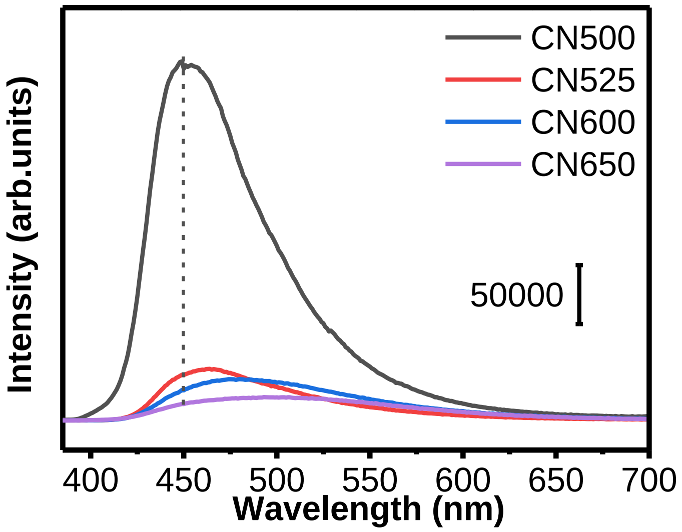

Supplementary Figure 3| Photoluminescence (PL) emission spectra, excitation wavelength: 365nm.

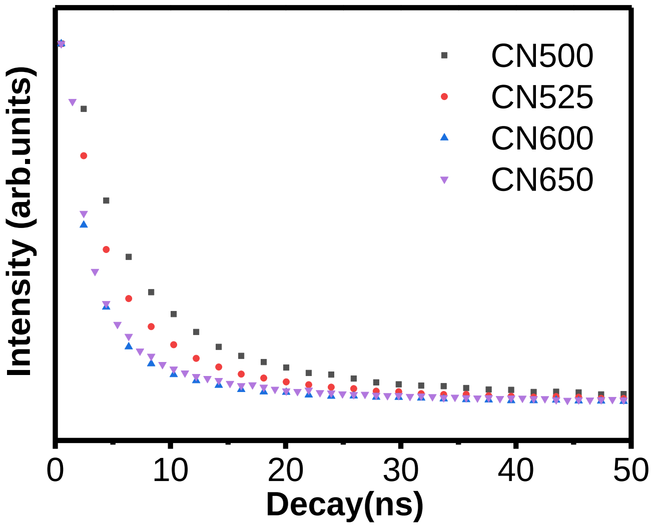

Supplementary Figure 4| Time-resolved PL decay spectra of carbon nitrides prepared at different temperatures.

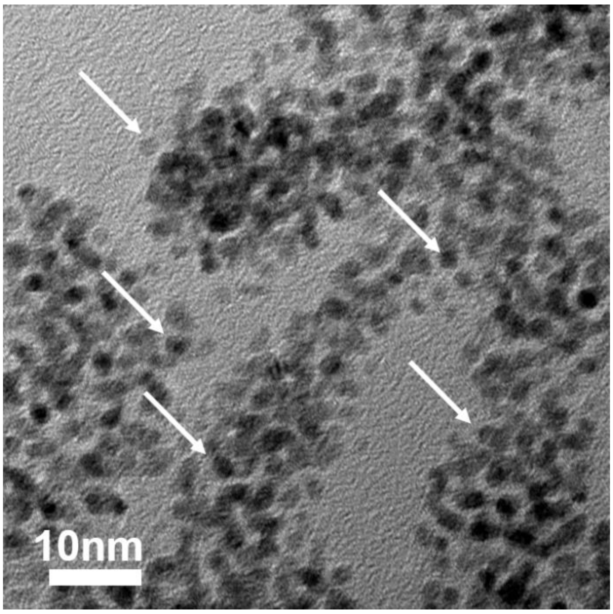

Supplementary Figure 5| TEM image of Pt nanoparticles in the colloid.

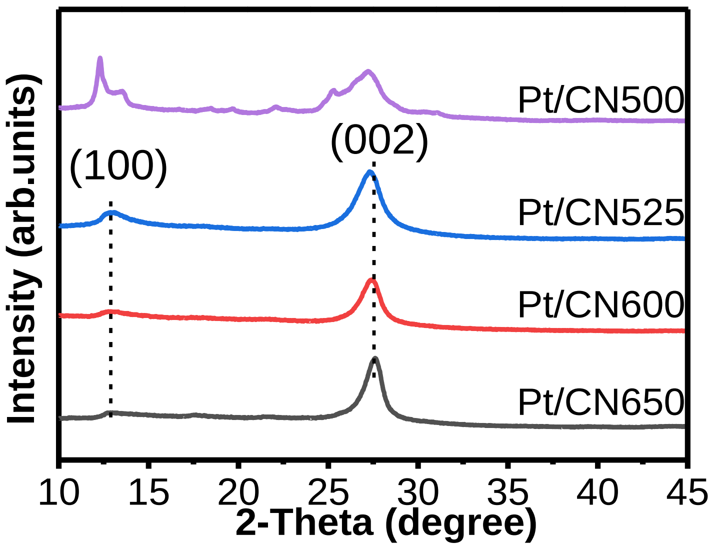

Supplementary Figure 6| XRD patterns of Pt supported on different carbon nitrides.

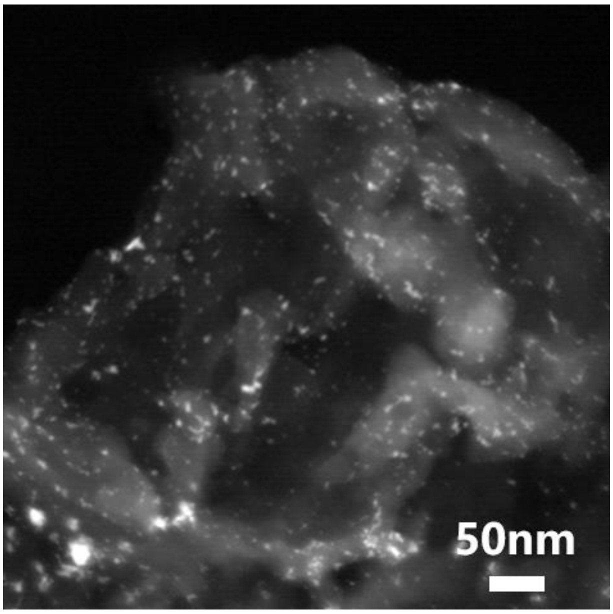

Supplementary Figure 7| HAADF-STEM image of 0.3wt% Pt/CN650.

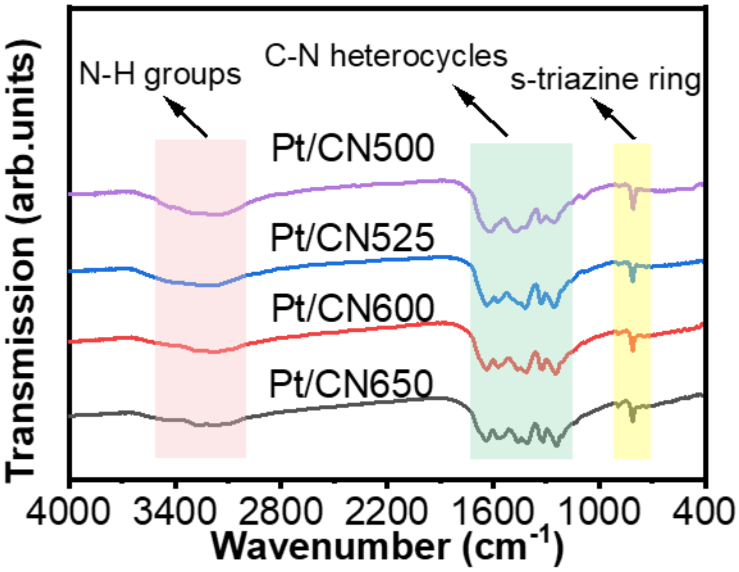

Supplementary Figure 8| FT-IR spectra of Pt supported on different carbon nitrides.

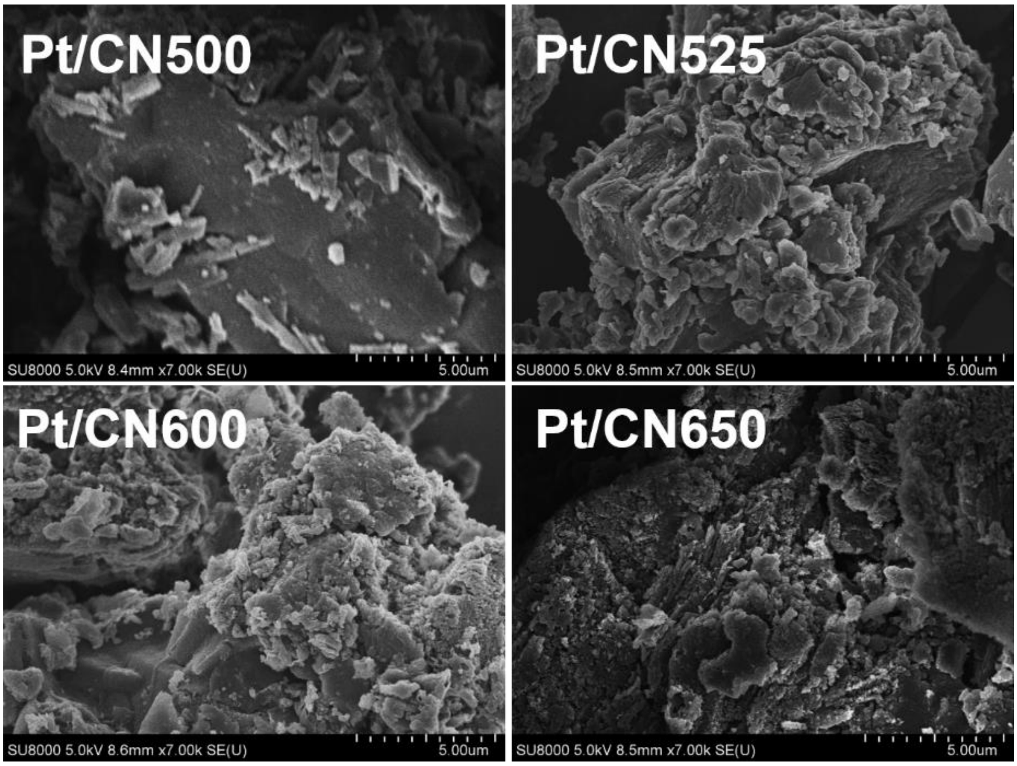

Supplementary Figure 9| SEM images of Pt supported on different carbon nitrides.

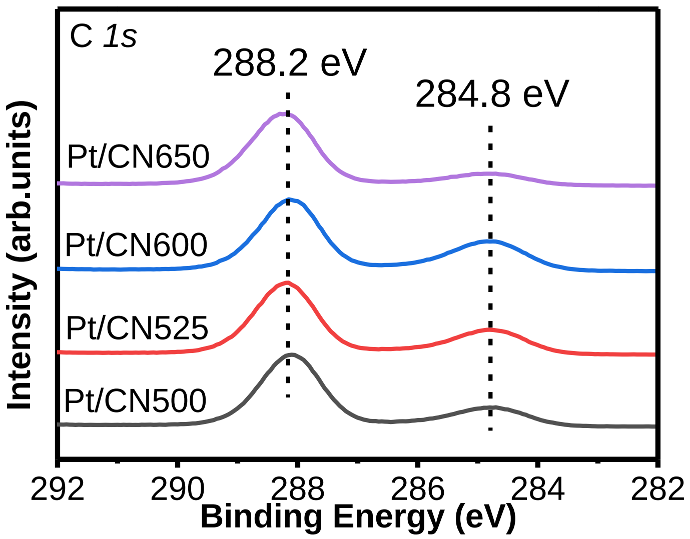

Supplementary Figure 10| C *1s* spectra of Pt supported on different carbon nitrides.

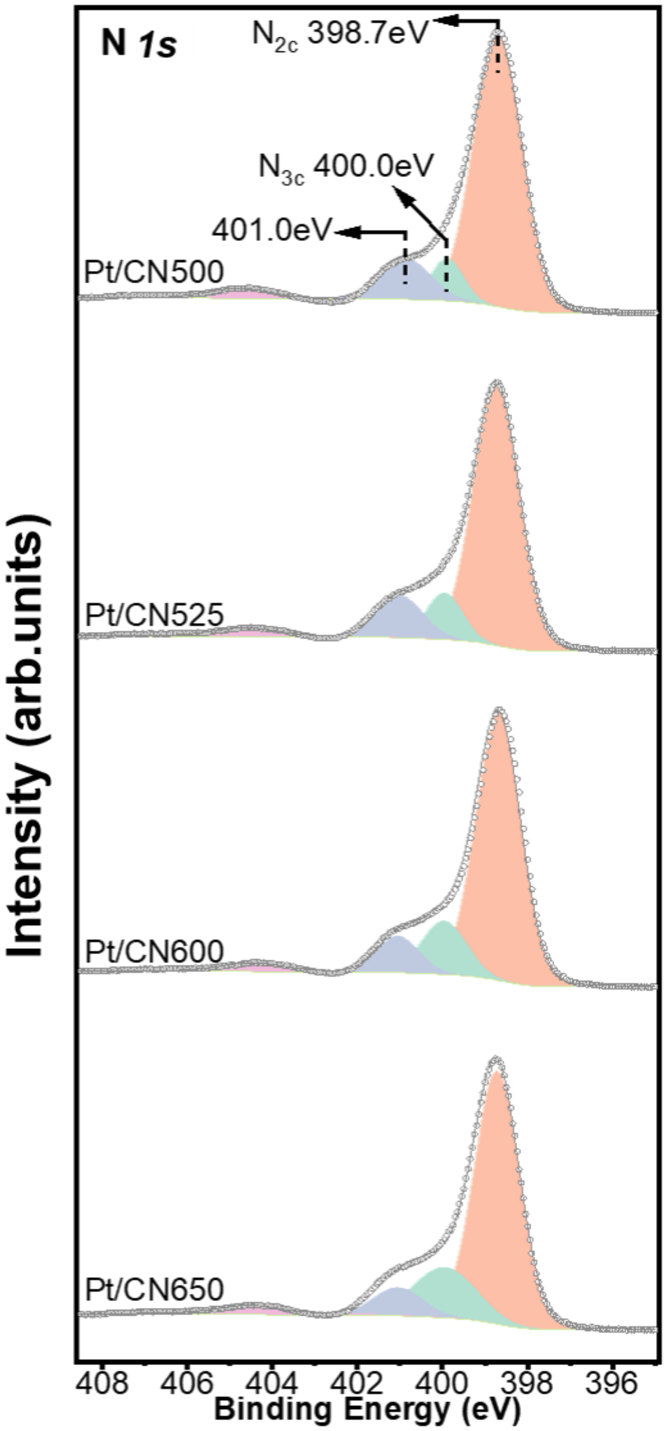

Supplementary Figure 11| N *1s* spectra of Pt supported on different carbon nitrides.

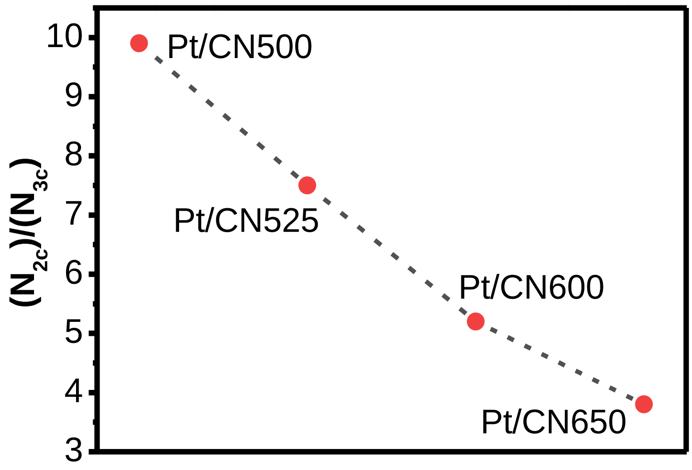

Supplementary Figure 12| The (N2c)/(N3c) ratio calculated by the high-resolution N *1s* spectra.

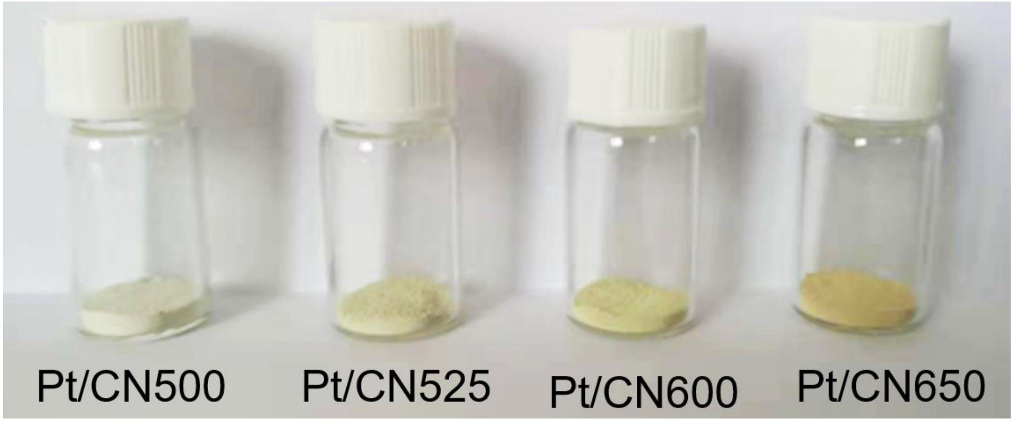

Supplementary Figure 13| Photographs of Pt supported on different carbon nitrides.

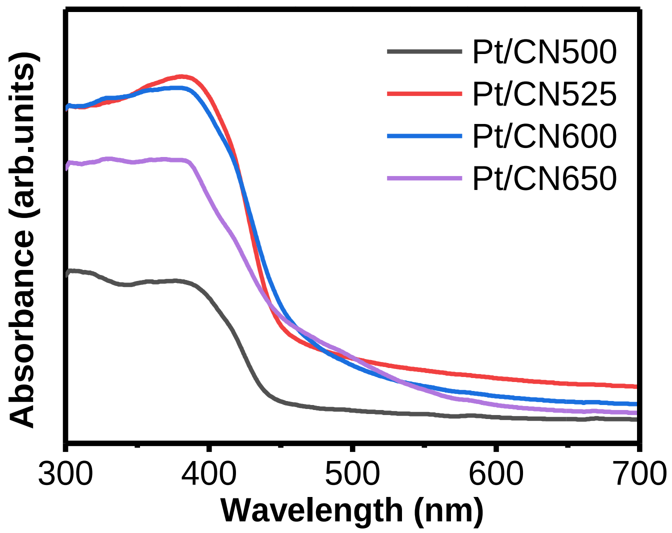

Supplementary Figure 14| UV-vis diffuse reflectance spectra of Pt supported on different carbon nitrides.

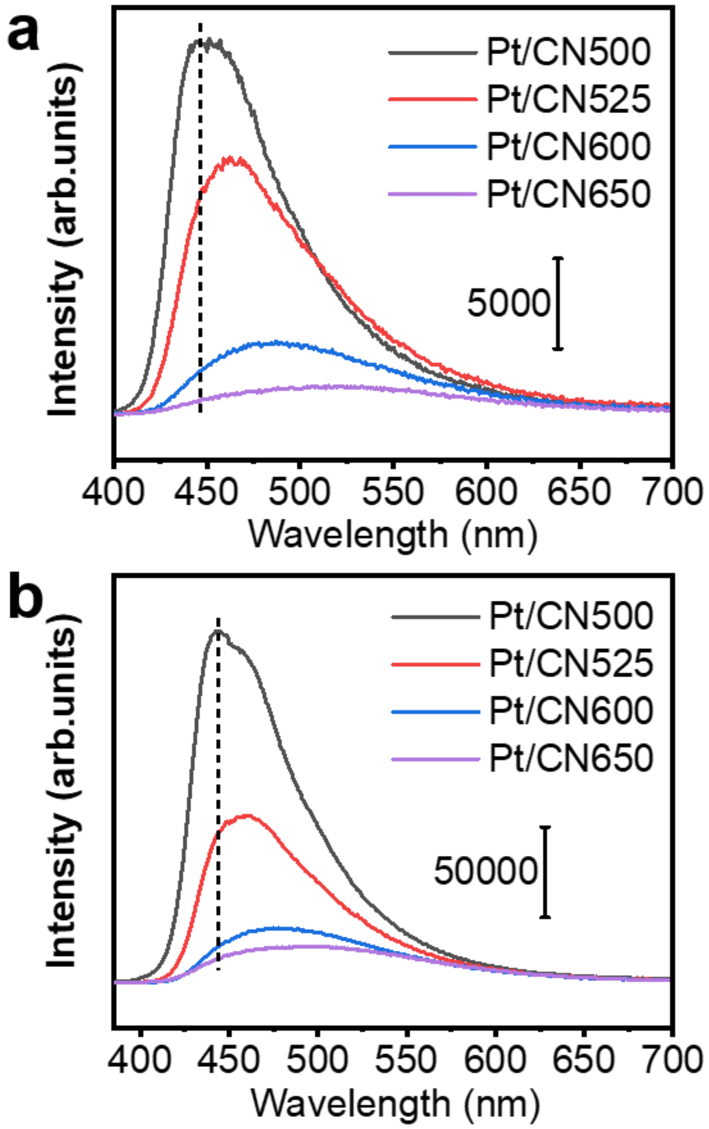

Supplementary Figure 15| Photoluminescence (PL) emission spectra, excitation wavelength: a 380nm, b 365 nm.

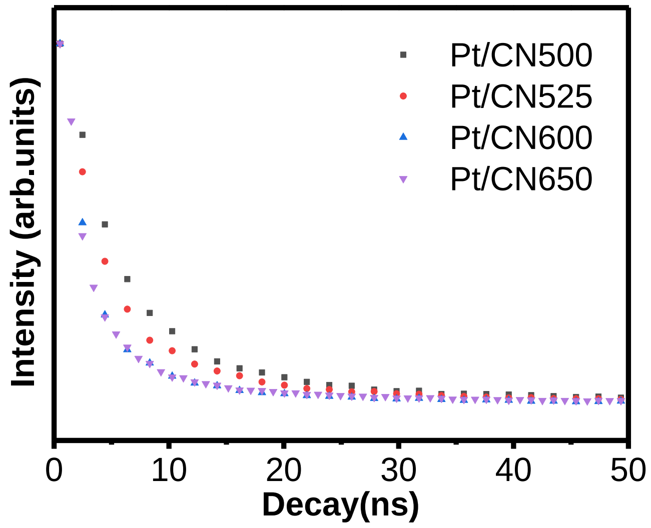

Supplementary Figure 16| Time-resolved PL decay spectra of Pt supported on different carbon nitrides.

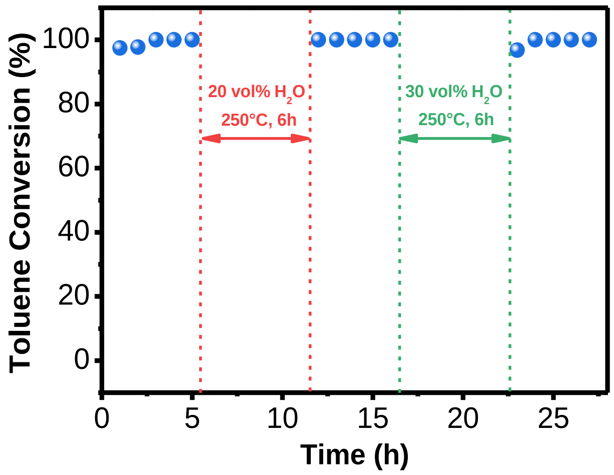

Supplementary Figure 17| Stability test of toluene oxidation over 0.3Pt/CN650 after different hydrothermal treatment.

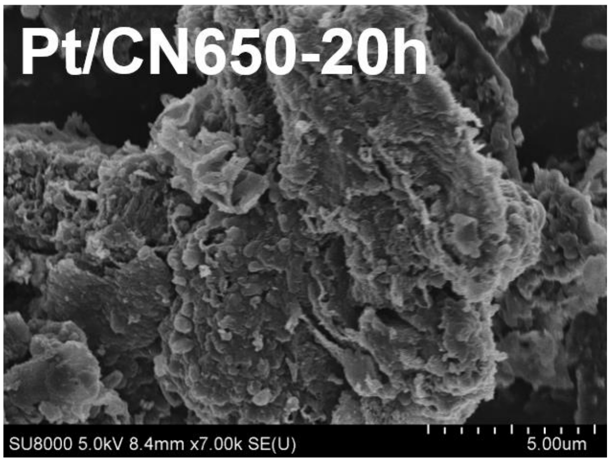

Supplementary Figure 18| SEM image of Pt/CN650-20h.

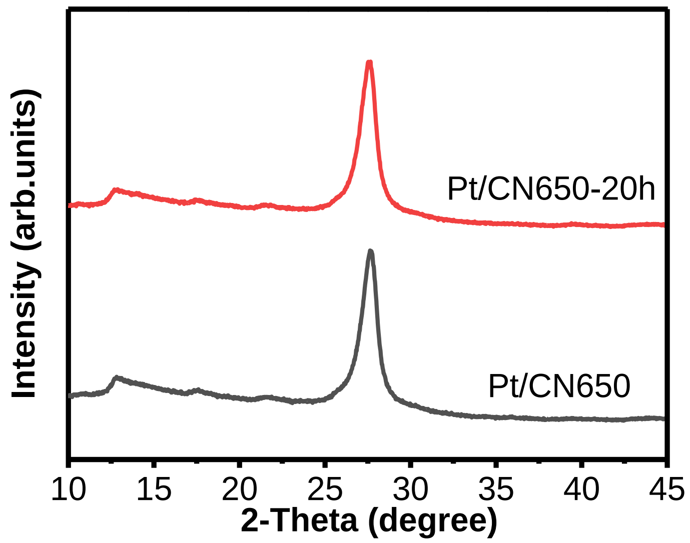

Supplementary Figure 19| XRD patterns of Pt/CN650 and Pt/CN650-20h.

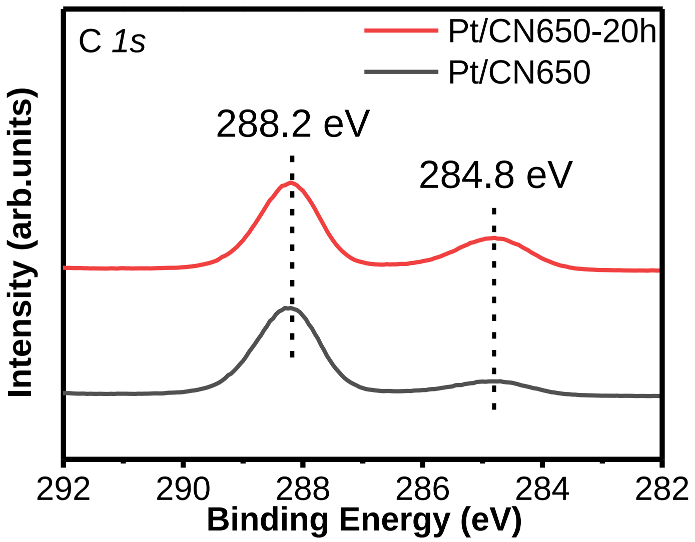

Supplementary Figure 20| C *1s* spectra of Pt/CN650 and Pt/CN650-20h.

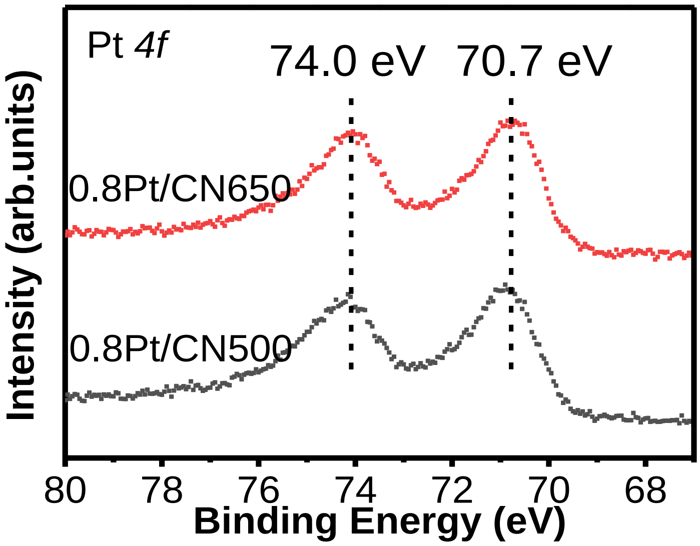

Supplementary Figure 21| Pt *4f* spectra of 0.8wt% Pt/CN500 and 0.8wt% Pt/CN650.

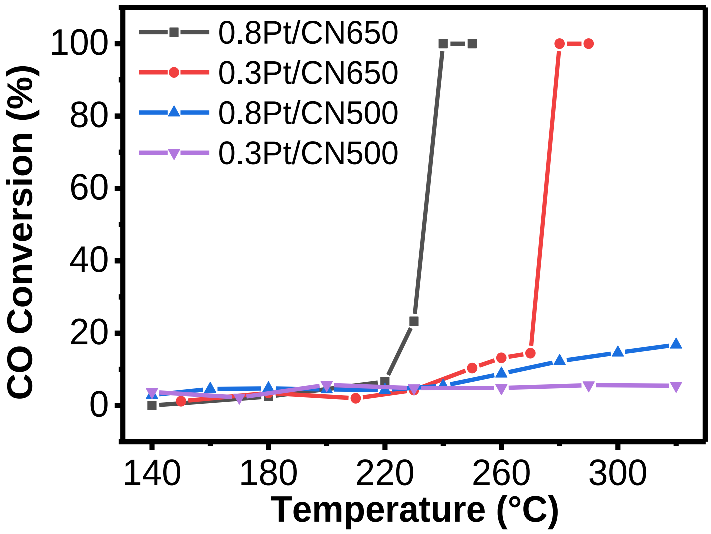

Supplementary Figure 22| CO conversion as a function of temperature over Pt/CN650 and Pt/CN500 with different loading amount of Pt, reaction conditions: CO 1 vol%, O_2 5 vol%, SV=75000 mL·g^{-1}·h^{-1}, Ar balance.

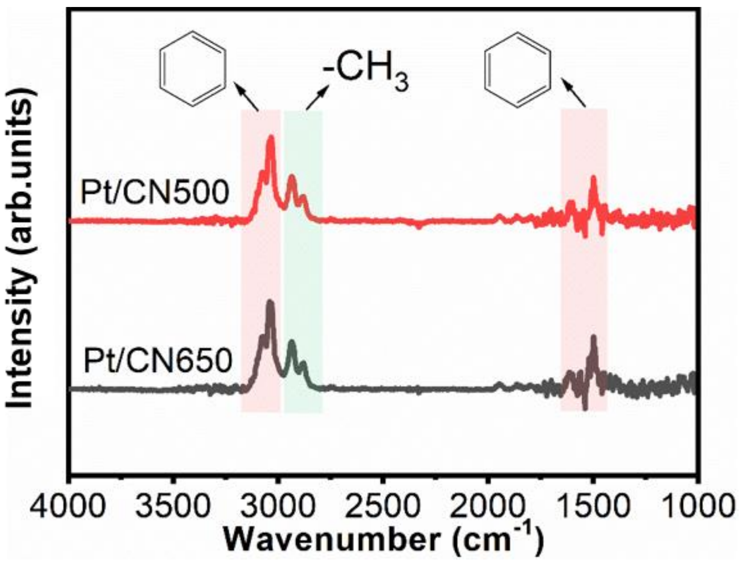

Supplementary Figure 23| In-situ C_7H_8-adsorbed DRIFT spectra of Pt/CN500 and Pt/CN650.

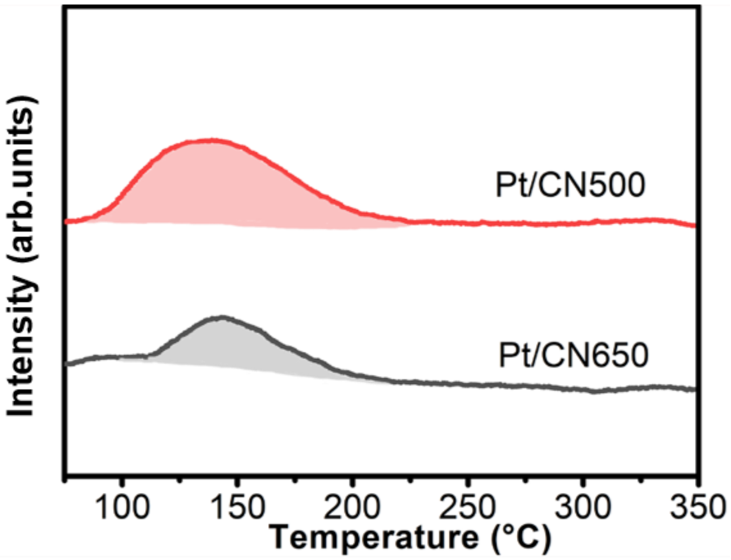

Supplementary Figure 24| C_7H_8-TPD profiles of Pt/CN500 and Pt/CN650.

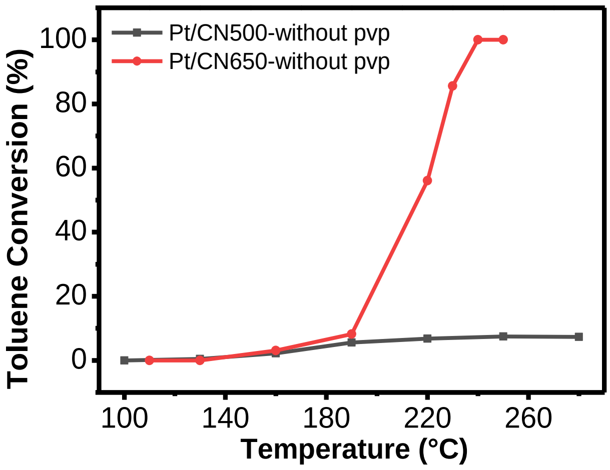

Supplementary Figure 25| Toluene conversion as a function of temperature over different samples.

Supplementary Tables

Supplementary Table 1| Texture properties of carbon nitrides prepared at different temperatures.

**samples**	**Area (m^2/g)**	**Volume (cm^3/g)**	**Pore Size (nm)**
CN500	6.3	0.04	24.3
CN525	7.1	0.04	18.9
CN600	25.3	0.10	16.5
CN650	31.6	0.15	19.4

Supplementary Table 2| Surface functionalities calculated based on peaks fit to XPS spectra.

Samples	N_{2c}(C=N-C)		N_{3c}(N-(C)$_3$)		C-N-H		Charge effects	
	eV	%	eV	%	eV	%	eV	%
**CN500**	398.8	76	400.0	9	401.0	11	404.5	4
**CN525**	398.6	75	400.0	11	401.0	9	404.5	5
**CN600**	398.6	71	400.0	16	401.1	8	404.5	5
**CN650**	398.6	69	400.0	20	401.1	6	404.4	5

Supplementary Table 3| The ratio of C to N calculated based on the XPS results.

**samples**	**C/N**
CN500	0.65
CN525	0.73
CN600	0.72
CN650	0.73

Supplementary Table 4| Emission decay lifetime of carbon nitrides prepared at different temperatures.

**Samples**	**τ_1(ns)**	**A_1(%)**	**τ_2(ns)**	**A_2(%)**	**τ_3(ns)**	**A_3(%)**	**τ (ns)**
CN500	5.5	46.8	29.4	29.9	264.3	23.3	226.9
CN525	4.2	51.9	22.2	33.0	170.8	15.1	129.5
CN600	2.8	52.4	15.8	33.2	116.2	14.4	86.7
CN650	2.1	41.1	12.0	38.8	95.6	20.1	76.7

Supplementary Table 5| Surface functionalities calculated based on peaks fit to XPS spectra.

**Samples**	**N_{2c}(C=N-C)**		**N_{3c}(N-(C)$_3$)**		**C-N-H**		**Charge effects**	
	eV	%	eV	%	eV	%	eV	%
**Pt/CN500**	398.7	77	399.9	8	400.9	12	404.8	4
**Pt/CN525**	398.7	75	400.0	10	401.0	11	404.6	4
**Pt/CN600**	398.7	73	400.0	14	401.1	9	404.5	4
**Pt/CN650**	398.7	69	400.0	18	401.1	8	404.6	5

Supplementary Table 6| Emission decay lifetime of Pt supported on different carbon nitrides.

**Samples**	**τ_1(ns)**	**A_1(%)**	**τ_2(ns)**	**A_2(%)**	**τ_3(ns)**	**A_3(%)**	**τ (ns)**
Pt/CN500	4.9	54.3	25.5	29.4	228.8	16.4	184.4
Pt/CN525	3.8	51.3	20.2	33.3	154.6	15.4	117.6
Pt/CN600	2.6	52.8	14.5	32.3	108.5	15.0	82.3
Pt/CN650	2.0	44.5	11.6	37.3	101.0	18.2	80.9

Supplementary Table 7| Catalytic activity for toluene oxidation over various catalysts

Catalysts	Toluene concentration (ppm)	space velocity (SV)	Completely conversion temperature (T_{100}, ℃)	Reference
**0.1%Pt/CN650**	1000	60000 mL • g^{-1} • h^{-1}	230	This work
**0.3%Pt/CN650**	1000	24000 mL • g^{-1} • h^{-1}	190	This work
**0.3%Pt/CN650**	1000	60000 mL • g^{-1} • h^{-1}	210	This work
**0.8%Pt/CN650**	1000	60000 mL • g^{-1} • h^{-1}	190	This work
**1%Pt/Beta-H**	1000	60000 mL • g^{-1} • h^{-1}	200	[1]
**0.78%Pt/HPMOR**	1000	60000 mL • g^{-1} • h^{-1}	210	[2]
**0.55%Pd/NiO**	500	19200 mL • g^{-1} • h^{-1}	210	[3]
**6.4%Au/LSMO**	1000	20000 mL • g^{-1} • h^{-1}	180	[4]
**3.26%Pd/CoAl-Al**	2000	60000 mL • g^{-1} • h^{-1}	230	[5]
**1%Pt/Al_2O_3-CeO_2**	1000	8400 mL • g^{-1} • h^{-1}	250	[6]
**0.89%Pt/CeO_2-AC**	1000	40000 mL • g^{-1} • h^{-1}	180	[7]
**0.5%Pt/TiO_2**	2000	34000 mL • g^{-1} • h^{-1}	250	[8]
**0.5%Pd/SC-10**	1000	32000 h^{-1}	210	[9]
**(0.77%Pt/CeO_2)-P**	200	50000 mL • g^{-1} • h^{-1}	230	[10]

Supplementary Table 8| Surface functionalities based on peaks fit to XPS spectra.

Samples	N_{2c}(C=N-C)		N_{3c}(N-(C)$_3$)		C-N-H		Charge effects	
	eV	%	eV	%	eV	%	eV	%
**Pt/CN650**	398.7	69	400.0	18	401.0	8	404.6	5
**Pt/CN650-20h**	398.7	70	400.0	17	401.1	8	404.5	4

Supplementary References

1 Chen, C. *et al.* Enhanced performance in catalytic combustion of toluene over mesoporous Beta zeolite-supported platinum catalyst. *Appl. Catal. B-Environ.* **140-141**, 199-205, (2013).

2 Zhang, J. *et al.* Enhanced toluene combustion performance over Pt loaded hierarchical porous MOR zeolite. *Chem. Eng. J.* **334**, 10-18, (2018).

3 Meng, Q. *et al.* In situ valence modification of Pd/NiO nano-catalysts in supercritical water towards toluene oxidation. *Catal. Sci. Technol.* **8**, 1858-1866, (2018).

4 Liu, Y. *et al.* Au/3DOM $La_{0.6}Sr_{0.4}MnO_3$: Highly active nanocatalysts for the oxidation of carbon monoxide and toluene. *J. Catal.* **305**, 146-153, (2013).

5 Zhao, S., Li, K., Jiang, S. & Li, J. Pd–Co based spinel oxides derived from pd nanoparticles immobilized on layered double hydroxides for toluene combustion. *Appl. Catal. B-Environ.* **181**, 236-248, (2016).

6 Abbasi, Z., Haghighi, M., Fatehifar, E. & Saedy, S. Synthesis and physicochemical characterizations of nanostructured Pt/Al_2O_3-CeO_2 catalysts for total oxidation of VOCs. *J. Hazard. Mater.* **186**, 1445-1454, (2011).

7 Abdelouahab-Reddam, Z., Mail, R. E., Coloma, F. & Sepúlveda-Escribano, A. Platinum supported on highly-dispersed ceria on activated carbon for the total oxidation of VOCs. *Appl. Catal. A-Gen.* **494**, 87-94, (2015).

8 Chen, X. *et al.* A facile route for spraying preparation of Pt/TiO_2 monolithic catalysts toward VOCs combustion. *Appl. Catal. A-Gen.* **566**, 190-199, (2018).

9 He, C. *et al.* Nanometric palladium confined in mesoporous silica as efficient catalysts for toluene oxidation at low temperature. *Appl. Catal. B-Environ.* **111-112**, 46-57, (2012).

10 Wang, B. *et al.* Effects of dielectric barrier discharge plasma on the catalytic activity of Pt/CeO_2 catalysts. *Appl. Catal. B-Environ.* **238**, 328-338, (2018).
